# Predictability Associated With Reduction in Phonetic Signals Without Semantics—The Case of Glossolalia

**DOI:** 10.1177/00238309231163170

**Published:** 2023-04-17

**Authors:** Samantha Link, Fabian Tomaschek

**Affiliations:** Deutscher Sprachatlas, University of Marburg, Germany; Department of General Linguistics, University of Tübingen, Germany

**Keywords:** Glossolalia, mental syllabary, smooth signal redundancy hypothesis, kinematic practice, lexical access

## Abstract

Glossolalia can be regarded as an instance of speech production in which practitioners produce syllables in seemingly random sequences. However, a closer inspection of glossalalia’s statistical properties reveals that sequences show a Zipfian pattern similar to natural languages, with some syllables being more probable than others. It is well established that statistical properties of sequences are implicitly learned, and that these statistical properties correlate with changes in kinematic and speech behavior. For speech, this means that more predictable items are phonetically shorter. Accordingly, we hypothesized for glossolalia that if practitioners have learned a serial pattern in glossolalia in the same manner as in natural languages, its statistical properties should correlate with its phonetic characteristics. Our hypothesis was supported. We find significantly shorter syllables associated with higher syllable probabilities in glossolalia. We discuss this finding in relation to theories about the sources of probability-related changes in the speech signal.

## 1 Introduction

### 1.1 Overview

The present study investigates glossolalia (or “speaking in tongues”), a case of articulatory production that is not based on processes, such as semantics, syntax, or morphology, which are typically attributed to natural languages. When practicing glossolalia, speakers produce seemingly random sequences of syllables. We tested whether the syllable sequences in glossolalia are truly random, or whether its practitioners implicitly learn some type of serial pattern as a result of practicing glossolalia. As it turns out, we did indeed find patterning rather than randomness. Analyzing the distributional properties of glossolalia—within the community and within individual speakers—we found that glossolalia syllables follow a statistical distribution similar to natural languages. As individual items in a serial pattern are predictable, and as predictability in speech correlates with phonetic properties, we hypothesized that if practitioners have learned a serial pattern in glossolalia in the same manner as in natural languages, its statistical properties should correlate with its phonetic characteristics. This is exactly what we have found. The statistical properties of glossolalia syllables correlate with their phonetic characteristics—with more frequent and more predictable syllables being shorter.

In the remainder of this article, we first introduce glossolalia and the context in which it is practiced. Next, we summarize the literature on serial pattern learning and discuss the literature on predictability in speech production and its association with phonetic variation. We present two analyses: In the first analysis, we discuss the statistical properties of glossolalia. In the second analysis, we investigate the degree to which the duration of glossolalia syllables correlates with the statistical properties of glossolalia. We conclude the study with a discussion about the sources of probability effects in glossolalia, considering different proposals regarding what stage of speech preparation represents the source of the probability effects found in natural languages. We finish by identifying some of this study’s implications for models of speech production and for pinpointing the locus of probability effects in speech planning.

### 1.2 Glossolalia

Glossolalia, also known as “speaking in tongues,” is a form of prayer, which is practiced mostly in the context of Christian charismatic communities during public services or as a personal language during private prayer (Bade, 2012; Samarin, 1972). Practitioners regard it as a supernatural manifestation proving the presence of the Holy Spirit (Hempelmann, 2004), referring to the description of Pentecost in Acts 2:4 (quoted from the King James Version of 1987):

And they were all filled with the Holy Ghost, and began to speak with other tongues, as the Spirit gave them utterance.

Furthermore, 1 Corinthians 14:2–19 considers glossolalia to be a special gift from the Holy Spirit known as charisma:

For he that speaketh in an unknown tongue speaketh not unto men, but unto God: for no man understandeth him; howbeit in the spirit he speaketh mysteries.

Accordingly, when a community member speaks glossolalia for the first time, this moment is understood as evidence of baptism with the Holy Spirit and as an individual experience with Pentecost that marks the entry into the charismatic community (Hempelmann, 2004).

Example (1) shows a transcription of one of the recordings in the current study.

(1) [jəʃʊaxɪadadadasɪɔnɔnɔsɔxjɔŋgɔdareanananaʃɪadadadaxɪɔrɔsɔ]

From a linguistic perspective, this utterance consists of a sequence of seemingly random syllables. The sequence does not contain any apparent word boundaries and no recognizable words, making it necessary to rely on breathing pauses when introducing transcription boundaries above the syllable level (Samarin, 1973). While glossolalia allows for complex syllable structures, the most frequent syllable structure is also the most basic possible, namely, CV.

Given its seemingly random nature, glossolalia has unsurprisingly attracted linguistic and psychological attention. Several investigations of glossolalia were conducted in the United States in the 1960s and 1970s, primarily focusing on how its structure could be best formalized and whether or not glossolalia should be considered as an instance of language.

For example, Samarin (1973) identified four formal features which characterize glossolalia: (1) echoism, that is, the repetition of similar syllable patterns with slight variations; (2) regularity of cadence, that is, only small variations in pitch; (3) a reduced inventory of sounds in comparison to the native language of the speaker; and (4) a preference for CV syllables, as shown in Example (1).

Enninger and Raith (1983) presented a more psycholinguistic account of glossolalia by arguing that the syllable structure of glossolalia is determined by some kind of underlying phonotactic model. They reasoned that this phonotactic model might be the statistical distribution of CV syllables in the speaker’s native language, or potentially a subset of the most frequent ones. Following Enninger and Raith’s (1983) account, we will first investigate the degree to which the syllable inventory of glossolalia overlaps with the inventory of practitioners’ native language, namely German in the case of our study.

Regarding the question of whether glossolalia can be considered to be a language, all researchers to date have in fact concluded that glossolalia is not a language (see also Bade (2012), who investigated other instances of “meaningless language”). Rather, it has been considered a unique psycholinguistic phenomenon that “theoretically should not be possible” (Motley, 1982), a case of “meaningless but phonologically structured human utterance, [. . .] bearing no systematic resemblance to any natural language, living or dead” (Samarin, 1972).

Some studies have considered whether glossolalia is produced in an altered state of mind. Goodman (1969) claimed that glossolalia is generated under the influence of a trance. This was, however, contradicted by Kavan (2004), who reported that glossolalia practitioners do not consider themselves to be in a state of trance (e.g., unlike yoga practitioners). This view is supported by Holm et al. (2011), who showed that speakers can start producing glossolalia whenever they choose, independently of their mental state. This was also the case in the current study.

Glossolalia also seems to correlate with higher mental stability. Kildahl (1972) found that glossolalia practitioners do not have “poorer” mental health and well-being than non-practitioners. On the contrary, Kildahl (1972), and more recently, Francis and Robbins (2003) have shown that practitioners of glossolalia have lower stress levels. Kildahl (1972) even considers glossolalia a psychological coping mechanism, observing that practitioners tend to be in a state of personal crisis and anxiety before their first time practicing glossolalia.

### 1.3 Effects of sequence learning

Independently of whether glossolalia is to be considered a language or not, it constitutes an instance of kinematic behavior during which its practitioners articulate sequences of syllables and therefore holds the potential to be a learned serial pattern. In the last couple of decades, numerous studies have used serial pattern learning experiments to investigate how humans learn sequences that follow some type of regularity.

To create test sequences, researchers typically employ stimuli with which they expect linguistic knowledge to interfere as little as possible. For example, Nissen and Bullemer (1987) used an experimental paradigm during which participants were presented asterisks in one of four positions, with the order of positions following a predictable pattern. In response to the presentation, participants had to press a key representing the location of presentation. Nissen and Bullemer (1987) found that participants’ key presses became faster as the experiment progressed, suggesting a strong learning effect.

The serial pattern paradigm has also been employed to investigate what type of feedback is required need to be provided to effectively learn patterns. Reber (1967) demonstrated that learners are capable of learning a serial pattern implicitly and without any instructions about underlying rules (see Ellis, 1994, for a review). This is further supported by Howard et al. (1992), who showed that a serial pattern can be learned by simple observation, that is, when no overt response is produced. Studies have also investigated the characteristics necessary for a serial pattern to be learnable. For example, Reber (1967) examined the effect of two sequence conditions: “grammatical,” that is, based on a simple finite-state grammar that created a fixed set of patterns, and “non-grammatical,” that is, random presentation that did not match a finite-state grammar. Participants presented with grammatical sequences produced fewer errors than participants presented with non-grammatical sequences, suggesting improved learning capabilities when sequences contain more (predictable) structure.

The presence of a structured serial pattern does not only affect how well participants learn a sequence, but also how they behave when responding. To this end, Cleeremans and McClelland (1991) presented dots in one of six locations on a screen and participants had to press an equivalent key. Cleeremans and McClelland (1991) found that key pressing latencies were shorter in response to “grammatical” sequences than randomized, “non-grammatical” sequences. They also found that transitional probabilities correlated with participants’ response times: When participants were presented with an asterisk in a more probable location given the previous stimulus, their response times were faster than when presented with an asterisk in less likely position, independent of whether a sequence was “grammatical” or not. These results demonstrated that participants learned probability distributions and used this knowledge to more quickly predict the location of an upcoming asterisk. Critically, studies show that people can learn a serial pattern and its underlying grammar independently of the modality of the stimuli—whether blinking asterisks or articulated syllables (Saﬀran et al., 1996). For example, Tomaschek et al. (2022) demonstrated that learning even occurs when participants have to type a sequence of letters, which are seemingly randomly presented. What is more, since they created a letter sequence for each participant, they demonstrated that learning happens on an individual basis. In the next section, we discuss how these findings on sequence learning relate to glossolalia.

### 1.4 The present study

Motley (1982) observed that the phones used in glossolalia seem to follow the distributional characteristics of a natural language. This is already visible in Example (1): some syllables, such as [da], [nɔ], and [na], seem to be more frequent than others. In our first analysis, we will therefore investigate whether this is just a tendency in our example, or whether indeed individual syllables as well as syllable sequences show a non-random distribution. Establishing that glossolalia syllables indeed follow a statistical distribution is important for our second analysis, as different frequencies and probabilities of glossolalia syllables suggest that practitioners have learned a particular probability distribution associated with glossolalia. Given that Cleeremans and McClelland (1991) found response latency to correlate with the statistical properties of asterisk locations, we might expect the statistical properties of glossolalia syllables to correlate in some way with their phonetic characteristics. Although no study so far has investigated the phonetic characteristics of glossolalia in relation to its statistical properties, we can look to the literature on probability effects in natural language production to guide our hypotheses.

Statistical properties in natural language production have typically been operationalized by measuring the frequency of occurrence of words, syllables, phones, and the transitional probabilities between them in the speech signal (e.g., Aylett & Turk, 2004, 2006; Cohen Priva, 2015). The general consensus is that as words, syllables, and phones become more frequent and more probable in the speech signal, their phonetic characteristics become more reduced. This reduction is manifest in shorter acoustic durations and the production of vowels nearer to the center of the vowel space. We thus expect exactly the same effect for glossolalia: When glossolalia syllables are found in contexts of higher probability, they should be shorter.

We did this by calculating the overall frequency and probability of syllables on the basis of the whole glossolalia corpus obtained for the present study. We regard this measure as reflective of potential community-wide knowledge of glossolalia. However, it is likely that individuals vary in their practice of glossolalia, which would be reflected in differences between speakers in terms of the syllables used and their statistical distributions. It is likely that these measures outperform statistical measures calculated on the basis of the entire community. This hypothesis is supported by studies that take individual learning experience into account when predicting behavioral characteristics (e.g., Falkauskas & Kuperman, 2015; Tomaschek et al., 2022). We tested this question in two ways. First, we investigated how strongly the glossolalia lexicons recorded in the current data overlapped across the speakers. Second, we contrasted how well the phonetic characteristics of glossolalia syllables were predicted by statistical measures gauged on the glossolalia corpus as a whole and statistical measures gauged for individual practitioners. Finally, productions of glossolalia are conceivably affected by a speaker’s native language in the same way that first languages generally affect the phonetic characteristics of second languages (Best et al., 2001; Casillas, 2019; Kartushina & Martin, 2019; Rojczyk, 2012, among many others). Accordingly, we tested to what degree glossolalia’s syllable duration can be predicted by frequency and probability estimates on the basis of a corpus of spontaneous speech in German. Our predictions were borne out. We found that glossolalia syllables were significantly shorter when they were more frequent and more predictable. In the remainder of this study, we first present the glossolalia data. We then present a regression analysis in which we show support for our hypothesis insofar as shorter syllable durations are associated with higher glossolalia predictability. We conclude the article by discussing the implications of the present results for models of speech production.

## 2 Material

The data set in the current study consists of 15 recordings of glossolalia, each with an average duration around 2 min, made by 11 different German native speakers (7 female; 4 male; ages 21–37). The longest recording (186 s) was provided by Speaker 10 (Main Recording) and the shortest (111 s) by Speaker 9 (Test Condition) (the durations and syllable-type and -token counts and the number of breathgroups for all recordings can be found in Table 1). All speakers belong to the same church in South-Eastern Germany and know each other. For the test-recordings, speakers were instructed to record glossolalia in the absence of all people, including the authors of this investigation. They were instructed to choose a place as quiet as possible to keep background noise to a minimum. To ensure relatively consistent recording quality, participants were instructed on how to use Praat or Audacity to perform the recordings. These instructions were made even more detailed for the main-recordings. However, as only four speakers could manage to do the main-recording, the test-recordings were included, as well because glossolalia data are very rare (Speaker 11 joined later and was directly given the instructions for the main-recording, thus, there is no test-recording for them). This was also the reason for the independent recording procedure: While phonetic experiments are typically performed under more highly controlled conditions, independent recordings presented several advantages. First, we were able to access a substantial number of glossolalia users, whose rarity has meant that previous studies have examined relatively few speakers at a time (Newberg et al., 2006: five participants; Samarin, 1972: one participant; Motley, 1982: one participant; Bryant & O’Connell, 1971: three participants; Bonfim, 2015: two participants; and Amanze & Shanduka, 2015: 20 participants). Second, independent recordings allowed practitioners to record themselves in a more natural environment than a laboratory.

**Table 1.** Duration (s) and Frequencies of Syllable Types, Tokens, and Breathgroups for All 15 Recordings.

Recording	Duration (s)	Syllable types	Syllable tokens	Breathgroups
Speaker 1 main	122	55	461	19
Speaker 1 test	124	74	520	19
Speaker 2 test	130	76	449	28
Speaker 3 main	150	142	731	31
Speaker 3 test	185	142	826	27
Speaker 4 test	129	70	499	33
Speaker 5 test	125	160	484	27
Speaker 6 test	133	58	471	28
Speaker 7 test	120	79	467	14
Speaker 8 test	121	92	486	47
Speaker 9 main	135	69	481	35
Speaker 9 test	111	55	381	23
Speaker 10 main	186	93	766	29
Speaker 10 test	131	110	397	19
Speaker 11 main	122	212	512	51

The audio data were manually segmented and annotated with X-SAMPA based on the German phonetic system using Praat (Boersma & Weenink, 1996). However, the assumption that glossolalia does not encode meaning is connected to difficulties segmenting glossolalic breathgroups into word-like units. Samarin (1972) has already been aware of this problem and notes: “One feels like dividing syllables /abc/ sometimes into /a bc/, sometimes into /ab c/, and so on.” Although some frequently reoccurring syllable sequences give the impression of potential words, it is actually not possible to segment them in a consistent manner. For this reason, segmentation was done only on the levels of syllables and breathgroups.

As can be seen in Table 1, practitioners use both lax and tense vowels in glossolalia. These vowels were transcribed with the German equivalent vowels. There were also instances of long and overly long vowels, which were marked for duration. Statistical analysis^1^ was performed in R (R Core Team, 2014).

## 3 *N*-gram analysis

### 3.1 General overview

In total, the present glossolalia data set contains 597 unique syllable types and 7,486 tokens. Table 2 shows the ten most frequent syllable *n*-grams (1–5). Rows are ordered in decreasing order of occurrence. [na] is the most frequent syllable, and iterations of [na] comprise the most frequent bigram, 3, 4, and 5 grams. At first glance, most of the syllables in Table 2 can be considered to be based on Standard German.

**Table 2.** Ten Most Frequent *n*-Grams of Whole Syllables and Their Number of Observations (Fr).

1 gram	Fr1	2 gram	Fr2	3 gram	Fr3	4 gram	Fr4	5 gram	Fr5
Na	722	na na	311	na na na	127	na na na na	44	na na na na na	13
Ra	485	la la	122	ra ra ra	48	ra ra ra ra	24	sʊ kʊ tʊ rʊ vʊ	13
La	361	ra na	98	la la la	45	na na va xan	16	kʊ tʊ rʊ vʊ xʊn	12
Ja	250	ra ra	93	ra na na	37	ja su to ro	16	ra ra ra ra ra	12
ba	230	na ja	85	ra na ja	32	sʊ kʊ tʊ rʊ	16	ra na na na na	11
da	229	ra ba	71	na na ja	30	ra na na na	16	na na na va xan	10
dɔ	185	da da	66	va xan dɔ	29	na na na ja	15	na na va xan dɔ	10
ka	182	ba ba	56	ba ba ba	27	sɔ trea na na	14	na ja su to ro	9
xa	147	va xan	48	trea na na	25	la la la la	13	ra ra ra va xan	9
ma	128	dɔ dɔ	47	rɪa la la	19	kʊ tʊ rʊ vʊ	13	na na na na ja	8

However, there are also syllables that are rare or even not present in Standard German, such as [rIa], [trea], and [xan]. Nevertheless, syllables with the diphthongs [Ia] and [ea] are typical for Bavarian as it can be seen in the following examples from the PhonD2-corpus (Lameli & Werth, 2021): [krɪak] *Krieg* “war” or [deaf] *(er) darf* “he is allowed to” or [vean] *werden* “to become” and also [xan] can be found in Ripuarian for *(er) kann* “(he) can.” As the speakers are from South Eastern Germany, the influence of Bavarian can be considered as quite likely.

To investigate to what degree the glossolalia lexicon was based on the practitioners’ native language, we compared the frequency distributions of the syllables in glossolalia and German. To do so, we calculated how often the glossolalia syllabic sequences occur in German using the Karl–Eberhards–Corpus of spontaneously spoken Southern German (KEC). The KEC contains 1-hr long dialogues between 40 pairs of German native speakers (Arnold & Tomaschek, 2016) with a total size of roughly 530,000 words (23,266 types). It provides force-aligned phone-level transcriptions for each word. During the frequency assessment of glossolalia sequences in German, word and syllable boundaries in the KEC were ignored. Pauses, breathing, and hesitations were interpreted as sequence boundaries. We found 381 of the glossolalia syllable types attested in the KEC (i.e., 60% of the glossolalia lexicon) and 216 types not attested in the KEC. When the test was performed on the basis of all glossolalia tokens, 90% of the glossolalia syllable tokens were attested in German.

So do practitioners simply mirror the German lexicon when practicing glossolalia? If so, then syllables in glossolalia should have the same frequency as in German and a high correlation between these two frequency distributions would be expected. However, the Spearman rank correlation between the glossolalia and German-type frequencies is ρ = .4 when tested on the entire data set, and ρ = .36, when only syllable types attested in both glossolalia and German are taken into account. Thus, the distributions of syllable frequencies are different in glossolalia and German. This difference in the statistical distribution implies that although some of the syllables are based on German, overall, these two lexicons may be relatively independent.

In addition to frequency, numerous studies have shown that the conditional probability of syllables, that is, the probability of a syllable appearing given the preceding or following syllable, also correlates with phonetic duration. Accordingly, we extracted the frequencies of syllable bigrams from the KEC. We find that our glossolalia corpus contains a total of 3,381 bigrams; 80% of the glossolalia syllable bigrams, that is, the majority of the lexicon, are not present in the KEC. The Spearman rank correlation between the bigram frequencies in glossolalia and German for syllables present in both languages is –.43.

Finally, previous studies have argued that glossolalia does not contain any proper words. It is nevertheless possible that practitioners borrowed some words from German and included them into their glossolalia lexicon. If this were the case, some glossolalia syllable bigrams should constitute full disyllabic words in the KEC. In total, 15 of the 3,382 glossolalia bigrams are full words in the KEC, demonstrating that this glossolalia corpus actually contains only a small number of real words. Among those are the high-frequency German words [daNk@] “thanks” and [hUnd@] “dogs.” Each of these words is present only once in the entire glossolalia data set. Most of the remaining words also occur only once, with a few ranging in frequency between 2 and 7 in the glossolalia corpus. These sequences may therefore have been articulated by chance rather than intentionally. The sequence fricative-V-n-d/t-V in particular has been previously reported as widespread in glossolalia (see Jakobson & Waugh, 2002; Luhrmann, 2012; Samarin, 1972), and indeed our corpus contains several forms like [xanda], [xIndʊ], and [hʊndə].

**Figure 1.**
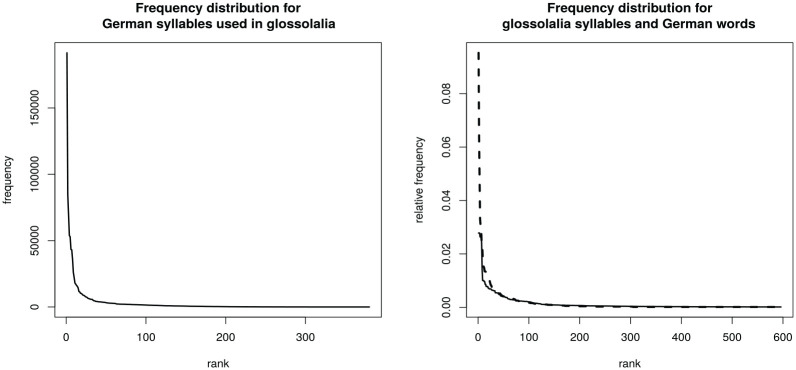
Left: Rank–frequency distribution of German syllables used in glossolalia. Right: Rank–frequency distribution of German words (solid line) and glossolalia syllables (dashed line).

Having established that the syllables in glossolalia show statistical variation (Table 2), we turn our attention to understanding to whether the frequency distribution of glossolalia syllables has language-like properties. Figure 1 (left panel) shows the distribution of German syllable frequencies for glossolalia syllables attested in German. The distribution follows a Zipfian pattern. There are a few very highly frequent syllables and a long tail with low-frequency syllables. Accordingly, unlike what Samarin (1972) reported for glossolalia in native speakers of English, the present instance of glossolalia was not based on a subset of the most frequent syllables in German. Instead, when practitioners sampled syllables from their native language’s lexicon, they sampled from the entire frequency range.

Following Zipf (1949), natural languages exhibit the distribution pattern illustrated in Figure 1 (right panel, solid line), which shows word counts of the top 597 words in German; we see a peak of high-ranked high-frequency words and a long tail for lower-ranked low-frequency words. Apart from the first-ranked entry, a similar distribution can be observed for glossolalia syllables (right panel, dashed line). This observation is supported by a Spearman rank correlation of ρ = .94 between the ranked glossolalia syllable frequencies and the ranked German syllable frequencies. In other words, glossolalia syllables follow the same statistical distribution as a natural language. Having established that the statistical properties of glossolalia syllables show characteristics of a natural language, we will inspect in the next section whether we can observe similar effects of statistical properties on glossolalia’s phonetic characteristics.

### 3.2 Speaker comparison

**Figure 2.**
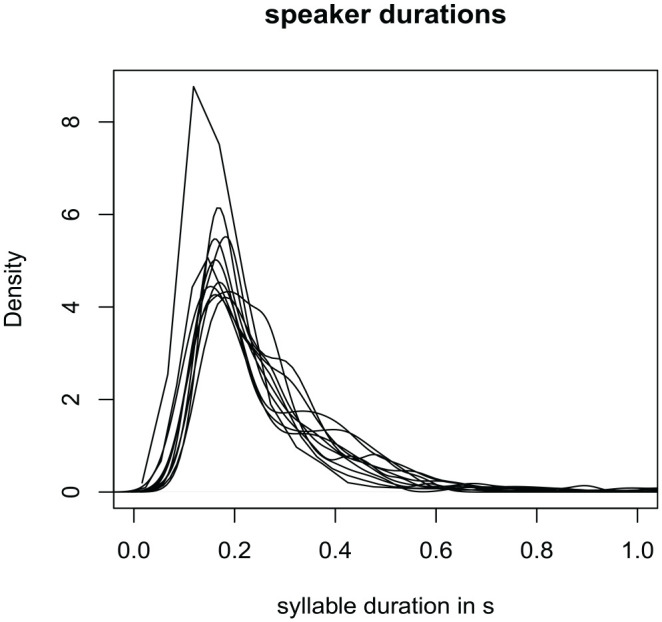
Density plots of syllable durations in glossolalia. Each line represents an individual speaker.

In the next section, we explore the variability of the glossolalia characteristics across speakers. We do so by comparing the distributions of syllable durations and the similarities and differences in the speakers’ glossolalia lexicons. Figure 2 illustrates that speakers show a strong overlap in the durations of the glossolalia syllables. The average overlap coefficient for a pair-wise comparison of the duration distributions is 0.85 (*SD* = 0.08) (calculated with the overlap function provided by the **bayestestR** package; Makowski et al., 2019). This high coefficient (1 indicates full overlap) indicates that speakers produce relatively similar durations.

**Figure 3.**
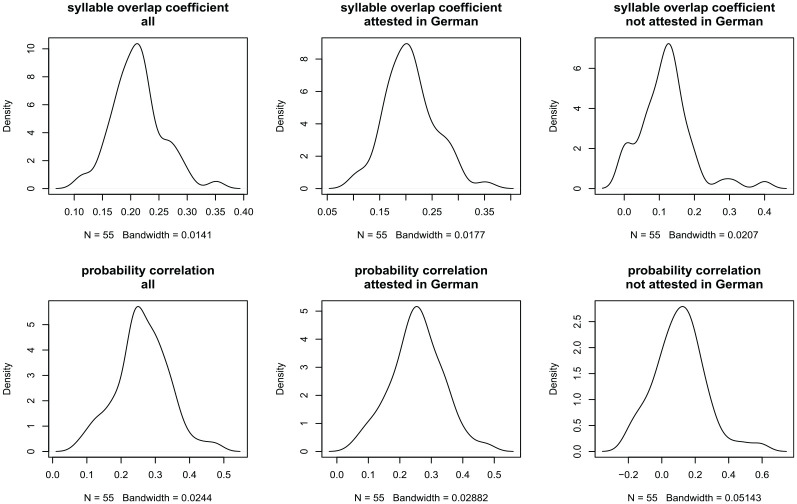
Top: Distributions of overlap coefficients gauging the number of shared syllables, calculated for all pair-wise speaker comparisons. Bottom: Distributions of Spearman’s rank correlations of syllabic probability of use between speakers. Columns represent comparisons for the entire glossolalia data set (left), those syllables that are attested in German (center), and those syllables that are not attested in German (right).

Next, we turn to the similarities among the speakers regarding their glossolalia lexicons. We operationalized the similarity among speakers by calculating the degree of overlap between speakers in a pair-wise comparison using the Szymkiewicz–Simpson coefficient. This overlap coefficient represents the amount of overlap between two sets in terms of percentages, calculated by dividing the number of syllables that intersect between two sets by the union of the two sets. A Szymkiewicz–Simpson coefficient of 1 indicates that one set is a perfect subset of another set. Figure 3 (top row) illustrates the distribution of overlap coefficients gauging the number of shared syllables, calculated for all pair-wise speaker comparisons. Overlap ranges between 0.1 and 0.4 for the entire glossolalia data set (left), with a peak at 0.2. Overlap for those syllables that are attested in German (center) ranges between 0.05 and 0.4, with a peak at 0.2. Finally, overlap for those syllables that are not attested in German (right) ranges between 0 and 0.4, with a peak at 0.1. Given that zero represents a lack of overlap between sets and one represents full overlap, these results indicate that there is only a small amount of overlap between practitioners in terms of the syllable types they use.

However, it is possible that practitioners share high-frequency syllables but differ in their use of low-frequency syllables, which would be reflected in a high correlation between practitioners’ syllabic probabilities. We tested this hypothesis by calculating pair-wise Spearman’s rank correlations of syllabic probabilities for all practitioners. Figure 3 (bottom row) indicates that we do not find such high correlations. While there are some pairs of practitioners whose glossolalia performances share statistical properties—indicated by correlations around .5 and .6—the majority of pair-wise correlations are low. In sum, practitioners show little overlap in their glossolalia syllables. This finding indicates that practitioners may actually not share a common lexicon but instead that each practitioner has their own glossolalia lexicon.

## 4 Analysis

### 4.1 Predictors

In this section, we turn our attention to the acoustic characteristics of glossolalia and to what degree they correlate with glossolalia’s statistical properties (the material and analysis can be inspected in the Supplementary material obtainable from https://osf.io/navp9/). To operationalize the statistical properties, we use two types of predictors, namely, syllable probability P(S0) (syllable frequency of occurrence divided by the size of the corpus) as well as three kinds of conditional probabilities: P(S0|S−1) , P(S0|S+1) and P(S+1|S0) , where S0 denotes the target syllable and S−1 and S+1 denote the syllable preceding/following the target syllable. Following Aylett and Turk (2004), Cohen Priva (2015), and Bell et al. (2009), we expect greater syllable probability and greater conditional probability to be associated with shorter syllable duration and greater vowel centralization.

We calculated these measurements on the basis of three data sets: First, on the basis of the entire glossolalia data set; second, on the basis of subsets based on the speakers; and third, on the basis of the KEC corpus of spontaneously spoken German (Arnold et al., 2017). Some glossolalia syllables were not present in the KEC, with a frequency of zero. To obtain transitional probabilities for these syllables, we performed add-one-smoothing by adding 1 to all frequency counts (Jurafsky & Martin, 2008).

Conditional probabilities on the basis of glossolalia were calculated as follows:

(1) $$P(S0|S-1)=\frac{P(S0,S-1)}{P(S-1)}$$

(2) $$P(S0|S+1)=\frac{P(S0,S+1)}{P(S+1)}$$

(3) $$P(S+1|S0)=\frac{P(S+1,S0)}{P(S0)}$$

Probabilities on the basis of the entire glossolalia lexicon were calculated by dividing bigram frequencies by the sum of all bigram frequencies. Individual glossolalia probabilities were calculated by dividing the frequency of individual syllables by the sum of all individual frequencies.

As all distributional measures showed a non-normal distribution with a long right tail, we log-transformed them to obtain quasi normal distribution. In addition, to be able to compare the size of the estimates, all predictors were *z*-scaled.

In the following results, statistical measures based on the entire glossolalia corpus are prefixed with “All,” those calculated for individual glossolalia practitioners with “Sp,” and those calculated on the basis of the German corpus with “Ger.”

### 4.2 Correlations among predictors

**Figure 4.**
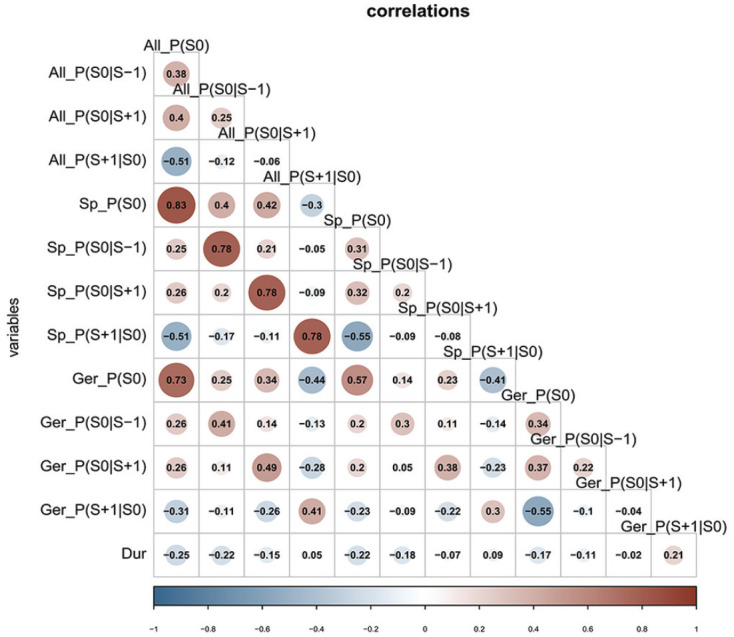
Spearman’s rank correlations among predictors used in the current study. *Note.* All = calculated on the basis of the entire glossolalia data set; Sp = calculated on the basis of glossolalia subsets for individual speakers; Ger = calculated on the basis of the KEC corpus for German. Bottom row shows the correlation between syllable duration (Dur) and the predictors.

Figure 4 illustrates the Pearson product-moment correlations among our predictors. Blue circles illustrate negative correlations and red circles represent positive correlations. We observe relatively high correlations between the measures obtained on the basis of individual data sets and their equivalents in the whole- and German-corpus measures. For example, Sp_P(S0) shows a high correlation with All_P(S0) and a moderate correlation with Ger_P(S0) . This has two implications. The first implication is that syllable probabilities based on the entire glossolalia corpus represent the syllable probabilities based on individual corpora relatively well. This may actually come as a surprise, given that there is only a small overlap between the lexicons of individual practitioners (cf. Figure 3). The reason for this seemingly contradictory behavior is that when there is little overlap between speakers, each of the speakers’ lexicons creates a cluster of syllables in the joint lexicon. When syllable probabilities are calculated in this joint lexicon, in the end, the clusters contribute stronger to the joint frequency count. By contrast, if there was a lot of overlap between speakers, then the individual speakers would form less clusters in the joint lexicon, and the correlation between individual probabilities and probabilities calculated on the basis of the entire glossolalia lexicon would drop. We demonstrated this type of relation between individual frequencies and accumulated frequencies in a simulation provided in the Supplementary material on OSF.

Second, the moderate correlation between probabilities in the glossolalia corpus and probabilities within the German corpus suggests that the statistical properties of glossolalia are not a one-to-one copy of German statistical properties.

The bottom line in Figure 4 illustrates the correlations between syllable duration in glossolalia and the predictors at hand. Note that all predictors apart from Ger_P(S0|S+1) and Ger_P(S+1|S0) show negative correlations with syllable duration in glossolalia.

### 4.3 Statistical model

Concerning the statistical analysis of glossolalia’s acoustic characteristics, the relatively high pair-wise correlations between the statistical measures (cf. Figure 4) could pose a severe problem if all predictors were added simultaneously to a statistical model. The reason is that collinearity among predictors can result in enhancement and suppression, that is, the erroneous increase or decrease of significance measures, such as *p*- or *t*-values, as well as changes of signs (Belsley et al., 1980). These effects may potentially result in uninterpretable coefficient estimates (see Tomaschek et al., 2018, for an illustration of collinearity’s effects on estimates). This is why a different modeling strategy is warranted: We fit each of these statistical measures individually in univariate analyses and compared their estimates, *t*-values, and the Akaike information criterion (AIC).

Specifically, we used linear mixed-effects regression provided by the R package *lme4* (Version 1.1-21, Bates, 2006), fitting syllable duration as function of one of the lexical predictors in a total of 12 models. We used speaker and syllable as a random intercept. For one predictor ( Ger_P(S0|S+1) ), we found that the inclusion of the syllable as random intercept changed the sign of the effect from positive to negative—a clear indicator of collinearity in the model. One potential reason for this effect is that 276 of the 573 different syllable tokens in the glossolalia data set were assigned an occurrence of 1. The high number of single tokens causes the random intercepts for syllable to explain a large portion of the variance that should be assigned to transitional probability as a fixed effect (see Baayen & Linke, 2019, for a detailed discussion of the effect of unique tokens). We therefore decided to fit all models with speaker as the only random effect. A comparison between the models with and those without syllable intercept indicated that the estimated effects of the individual predictors remained relatively similar between models with and without syllable as a random intercept (apart from Ger_P(S0|S+1) ). This was also the case for the t-values, even though these were smaller when random intercepts were included. Moreover, no predictor lost significance when syllable was included as a random effect. We are therefore confident that the models without syllable as random intercept are valid, which is why we present these in the present study. Quantile–quantile plots of theoretical and sampled residuals did not indicate any overly strong outliers, which is why we did not exclude any residuals from the fit.

### 4.4 Results

**Figure 5.**
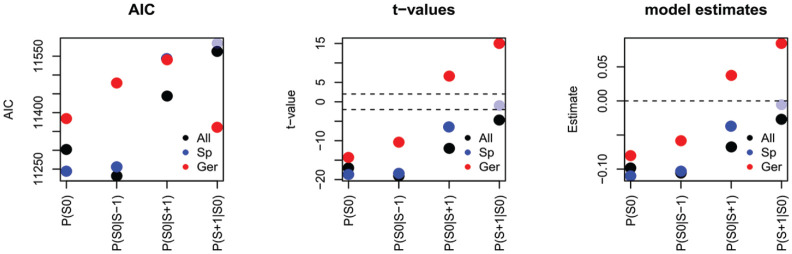
AICs (left), *t*-values (center), and estimates (right) of univariate models fitting glossolalia syllable duration. The x-axis represents the statistical measure. Pale dots indicate that the estimate was not significant. *Note.* Measures obtained on the basis of the entire glossolalia data set = All (black); on the basis of glossolalia data sets for individual speakers = Sp (blue); on the basis of the German KEC corpus = Ger (red).

Figure 5 illustrates the AICs (left), *t*-values (center), and estimates (right) of all univariate models. Keeping in mind that better model fits are mirrored by lower AICs, we find that all statistical measures based on German, apart from Ger_P(S+1|S0) , yield worse model fits than measures based on glossolalia. P(S0) yields a better model fit when calculated for individual practitioners than on the basis of the entire glossolalia data set. For the other statistical measures, this is reversed, that is, measures on the basis of the entire glossolalia data set yield a better model fit than those calculated on the basis of individual data sets.

Turning our attention to the *t*-values, we find that apart from P(S+1|S0) calculated on the basis of individual data sets (pale blue dot), all predictors yield |t|>2 , indicating that they are significant.

Recall that negative estimates indicate shorter syllable duration associated with greater statistical measures. Accordingly, the estimates indicate that shorter syllable duration in glossolalia is significantly associated with higher syllable probabilities and conditional probabilities, when these statistical measures are calculated on the basis of glossolalia. We also observe negative effects for P(S0) and P(S0|S−1) when calculated on the basis of German.^2^

Having compared the different sources of our probabilistic measures, we turn our attention to comparison of the strength of the effects. We first focus on the measures calculated on the basis of the entire Glossolalia corpus. We observe that P(S0) and P(S0|S−1) yield lower AICs, larger absolute *t*-values and stronger effect sizes than P(S0|S+1) and P(S+1|S0) . This difference in the strength of the effects is relatively similar for the measures calculated on the basis of the speakers and on the basis of German. To understand this effect, we need to discuss what these measures represent in the speech production process. P(S0) and P(S0|S−1) can be considered to represent the previous planning process of the syllable currently articulated, while P(S0|S+1) and P(S+1|S0) represent the planning of upcoming syllables while the current one is articulated. Taking this consideration into account, and given the difference in effect size between these predictors, it follows that planning the articulated syllable has a stronger effect on phonetic characteristics than the planning of upcoming syllables.

Taken together, these findings support our hypothesis that phonetic characteristics of glossolalia should mirror the effects of predictability on segment, syllable, and word duration in natural languages (Aylett & Turk, 2004; Bell et al., 2009; Cohen Priva, 2015). In contrast to our expectations, P(S0|S+1) and P(S+1|S0) calculated on the basis of German yield positive effects, indicating that the positive correlations between duration and these two predictors observed in Figure 4 are indeed statistically significant. Given the data at hand, we do not have any explanation for this effect.

In conclusion, we find that statistical properties of glossolalia correlate with the duration of glossolalia syllables. Specifically, we observe that higher probability is associated with phonetic reduction in the temporal domain.

## 5 General discussion

In the present study, we investigated glossolalia, a form of prayer during which practitioners produce seemingly random sequences of syllables. Glossolalia can be considered an instance of speech production that is not based on processes typically attributed to languages, such as semantics, syntax, or morphology, which is why it cannot be regarded as a natural language. Nevertheless, we demonstrated that its statistical properties are similar to natural languages. Some glossolalia syllables are more frequently used than others, which is why they vary in their global and local probability.

Based on studies of sequence learning (Cleeremans & McClelland, 1991; Howard et al., 1992; Nissen & Bullemer, 1987; Reber, 1967; Saﬀran et al., 1996; Tomaschek et al., 2022), we hypothesized that practitioners of glossolalia have learned the statistical properties and distributions of glossolalia. Following studies that have demonstrated how statistical properties correlate with phonetic variation in natural languages (Aylett & Turk, 2004, 2006; Bell et al., 2009; Cohen Priva, 2015; Meunier & Espesser, 2011), we expected to observe the same effects for glossolalia. Our hypothesis turned out to be supported. We found that higher probability, measured by syllable probability and conditional probability, was significantly associated with shorter glossolalia syllables.

In the following sections, we discuss the implications of our findings concerning the relationship between glossolalia and the practitioners’ native language. We also discuss how these effects can be explained given current theories regarding the function of probability in speech production.

### 5.1 Relationship between glossolalia and the practitioners’ native language

Let us first discuss the nature of glossolalia. Samarin (1972) found that the glossolalia syllables of native speakers of English consisted of a subset of English syllables. In our study, we also found that roughly 60% of the types and roughly 90% of the tokens in our glossolalia corpus can be found in German. The remaining tokens not represented in German constitute syllables, such as [SIa, trea, or xan] (with the first and second containing a diphthong), indicating that practitioners create new syllables for glossolalia that do not follow German phonotactics (see the appendix for more information).

We found another similarity between glossolalia and the practitioners’ native language. The rank–frequency distribution in glossolalia shows a Zipfian distribution, just as in German (see Figure 1). Numerous studies have demonstrated that the lexicons of natural languages show this Zipfian distribution (at least when the aggregated lexicon is considered; see Linke & Ramscar, 2020). Given that syllabic probabilities show a difference in frequency of use between glossolalia and German—as indicated by the small correlation between glossolalia and German syllable types ( ρ = .43 for the entire data set, ρ = .36 for syllable types only attested in German)—the observed Zipfian distribution indicates that glossolalia syllables form a lexicon of their own. Probabilities calculated on the basis of German also have a weaker effect on glossolalia’s phonetic characteristics than the glossolalia lexicon does—as evidenced by larger AIC values and smaller beta estimates when using the German probability measures—indicating that the glossolalia lexicon exists in addition to the lexicon of the practitioners’ native language.

The question of how this glossolalia lexicon could have developed thus arises. Here, we can only hypothesize. We assume that when practitioners begin to practice glossolalia, they start with a small set of syllables randomly sampled from the entire set of German syllabic sequences—most probably simple CV syllables that are frequent in German. With every repeated use of glossolalia, speakers keep borrowing syllables from their native language. It is possible that practitioners experiment with new syllables unknown in their native language right from the start. Furthermore, practitioners may share syllables among each other as they observe performances of glossolalia in their community. However, the amount of shared material is relatively small in the present data set. Moreover, at some point, the frequency distribution of glossolalia syllables diverges from that of German, along with the transitional probabilities between syllables. These measures suggest that practitioners’ syllable sets remain relatively independent from each other, as well as independent from German. If these assumptions are correct, glossolalia actually provides a solid platform for studying the development of lexicons. The conclusion that practitioners developed individual lexicons contrasts with our finding on the differences in goodness of fit of individual probability measures and of those estimated on the basis of the entire glossolalia data set. Specifically, conditional probabilities yielded a worse goodness of fit when predicting syllable duration when they were calculated for individual practitioners than when they calculated on the basis of the entire glossolalia data set. Only frequencies of occurrence for individual practitioners performed better than frequency of occurrence based on the entire data set. A potential reason for why this might be the case is that the different types of measures differ in their distributions. We tested this by comparing the distributions of P(S0) and P(S0|S−1) based on individual practitioners (Sp) and of those measures based on the entire data set (All) to a simulated normal distribution. And indeed, a Pearson-product moment correlation test indicated that Sp_P(S0) shows stronger resemblance with a normal distribution than All_P(S0) , while All_P(S0|S−1) has a more normal distribution than Sp_P(S0|S−1) . Thus, the differences in the goodness of fit very likely arise from the differences in distributions.

This brings us to the next research question in the present investigation: How is glossolalia affected by the practitioners’ native language, German?

### 5.2 Effects of German on glossolalia

Even though the token frequency between German and glossolalia is relatively low, indicating that the lexicons of both seem to be independent, lexical measures on the basis of both “languages” were significantly correlated with syllable duration in glossolalia. This effect indicates that both lexicons simultaneously affect articulatory processes in each syllable and that the motor programs of glossolalia are not completely independent of German lexical structures. This observation parallels findings that native languages affect the characteristics of both perception (Best et al., 2001) and production (Casillas, 2019; Kartushina & Martin, 2019; Rojczyk, 2012) of second languages. It is thus possible that glossolalia has reached the status of a “second language” for each of the practitioners.

What about effects in the reverse direction, then? Glossolalia appears to have strong effects on its practitioners’ cognitive processes, as demonstrated by the finding that practicing glossolalia improves the detection of new grammatical structures (Kéri et al., 2020). The statistical properties of the glossolalia lexicon may thus interact with the phonetic characteristics of practitioners’ native language, much the same way that second languages have been demonstrated to affect pronunciations in one’s native language (Bergmann et al., 2016; Harada, 2003). As longer glossolalia syllables are associated with greater German syllable conditional probabilities, this effect is inhibitory. Thus, German syllables with a high probability hinder practitioners to articulate their glossolalia properly.

Another question still remains: What is the source of the correlation that we observe between statistical properties and glossolalia’s phonetic characteristics? We discuss potential explanations in the next section.

### 5.3 The source of the probability effect

We have found that higher probability is associated with shorter durations of glossolalia syllables. Numerous studies have reported these kinds of phonetic effects in natural languages (e.g., Bell et al., 2009; Gahl, 2008; Landauer & Streeter, 1973; Lohmann, 2018; Tremblay & Tucker, 2011; Whalen, 1991; Wright, 1979), interpreting them as indicative of phonetic reduction. What mechanism is most likely responsible for these effects in glossolalia? To answer this question, we first discuss what explanations have been proposed for natural languages, and to what degree they are applicable to glossolalia. The explanations for natural languages can be roughly grouped into two perspectives: a listener- and a speaker-oriented perspective.

Studies favoring the listener-oriented perspective have argued that the association between acoustic characteristics and predictability reflects the amount of information conveyed in the speech signal (Aylett & Turk, 2004, 2006; Brandt et al., 2019; Cohen Priva, 2015; Hall et al., 2018; Jaeger, 2010; Le Maguer et al., 2016; Malisz et al., 2018; Schulz et al., 2016). Following information theory (Shannon, 1948), probability measures are assumed to assess the amount of information conveyed by the signal, with higher probability being associated with less information. Sudden changes in the flow of information between speaker and listener increase processing costs on the part of the listener. Thus, to avoid spikes in the flow of information, the acoustic complexity of the phonetic signal is modulated in relation to probability, and thus also to information. The duration and acoustic complexity of more predictable, less informative items is reduced, while more informative items are acoustically enhanced. As a result, the average flow of information per time unit is considered to be more uniform. Jaeger (2010) has argued for a general mechanism that monitors the flow of information, presented as the uniform information density hypothesis (see also Frank & Jaeger, 2008). Aylett and Turk (2004) have presented an alternative explanation within the listener-oriented perspective, arguing for a language-inherent mechanism that signals changes in information—namely, prosody. From this perspective, information density is mediated via prosodic prominence, with less informative words being acoustically less salient.

Are the two explanations from a listener-oriented perspective applicable to glossolalia? It seems that glossolalia does not convey any meaning—at least not in the material recorded for our study. This assumption is supported by our finding that speakers share only a small number of syllables. Also, one might hypothesize that as there is no meaning involved, practitioners are not directly involved in any communicative act. Accordingly, there is probably no need to manage the flow of information between speakers and listeners. Moreover, even though the sequence of syllables seems to be governed by some probabilistic system, it lacks any kind of abstract syntactic structure to which prosodic structures could be allocated. On the basis of these considerations, it follows that the listener-oriented perspective proposed for natural languages—along with its information-theoretic explanation of the relationship between probability and phonetic variation—cannot explain the probability effects in glossolalia.

From a speaker-oriented perspective, three explanations have been put forth. The first explanation focuses on the lexical stage of speech production. In all models of speech production and preparation (e.g., Baayen et al., 2019; Caramazza, 1997; Dell, 1986; Foygel & Dell, 2000; Levelt et al., 1999; Roelofs, 1997), production is initiated at the semantic level; semantic material activates and selects further lexical information related to a word’s syntax and morphology. Some researchers argue that more probable words are more readily available in the mental lexicon during this process (Bell et al., 2009; Gahl, 2008). Faster availability allows subsequent cognitive preparation processes to be executed faster, which in turn gives rise to faster articulatory speed, resulting in shorter acoustic durations and more reduction. However, this explanation may also not be applicable to glossolalia. As glossolalia does not convey any meaning, it is unlikely to be initiated by semantic material activating subsequent cognitive processes. Thus, while lexical processes are not the solution, post-lexical processes might be.

To produce glossolalia syllables, practitioners need to retrieve some kind of representation of articulatory gestures. Such representations may potentially be obtained from the “mental syllabary,” a module of speech preparation that is suggested to contain pre-stored motor programs for syllables (Levelt et al., 1999). In natural languages, it has been shown that more frequent syllables yield shorter production latencies than less frequent syllables. By contrast, less frequent syllables require longer preparation time (Buerki et al., 2015; Cholin et al., 2006; Cholin & Levelt, 2009). Based on these temporal differences, it has been argued that the syllabary contains motor programs for frequent syllables while rare syllables are assembled anew each time they are produced. The speed of access to syllabic motor programs may thus affect articulatory pace just like faster or slower lexical access does (Bell et al., 2009; Gahl, 2008); in turn, articulatory pace should correlate with modulations of temporal characteristics (Gay, 1977, 1978; Lindblom, 1963).

How can we apply this perspective to the effects of probability in glossolalia? Two mechanisms are actually possible. First, Levelt and colleagues have argued that the mental syllabary serves to transform abstract phonological sequences—which are the result of preceding lexical and morphological processes—into articulatory movements. Let us therefore assume that abstract phonological sequences are necessary to obtain motor programs from the syllabary. In this case, glossolalia would be based on creating random phonological CV (or more complex) sequences. Once these are syllabified, syllabic motor programs are selected from the syllabary. When syllables are not immediately available in the syllabary because they are rare, new motor programs are created for them. This difference in availability would then be mirrored in phonetic variation. The second mechanism involves the possibility that no sequence of abstract phonemes is involved during the articulation of glossolalia. Instead, practitioners randomly select syllables from a set of stored syllabic motor programs. In this case, the syllabary would contain motor programs for all existing syllables independently of their frequency. As more frequent syllables have a higher baseline activation than less frequent syllables (equivalent to proposals by Dell, 1986) they are selected faster for articulation than those with a lower baseline activation. Stronger baseline representation may emerge from a more frequent articulatory practice, as suggested by Tomaschek et al. (2018); Tomaschek et al. (2020). Only completely new glossolalia syllables, that is, those that speakers have never articulated before, will rely on some kind assembling routine that creates new motor programs for them. These differences in baseline activation, along with the differences in selection time, result in the articulatory and phonetic variation discussed above. Recall that probabilities calculated for both German and glossolalia correlated with the acoustic characteristics of glossolalia syllables, supporting the assumption that these two actually share a syllabic representation.

Another explanation from the speaker-oriented perspective focuses on the kinematic principles of human behavior and is rooted in general biological and physical constraints relating to the reduction of effort (Hoyt & Taylor, 1981; Lebedev et al., 2001; Zipf, 1935). These constraints dictate that longer articulations require more articulatory effort than shorter articulations. As the repetition of longer articulations would require in total more effort than the repetition of shorter articulations, frequent productions become shorter to reduce the total amount of effort. This perspective requires that speakers possess some kind of knowledge about the frequencies of syllabic occurrence. If this is the case, it could explain the effects of probability in glossolalia. Practitioners simply want to save effort when they articulate more frequent (and thus more probable) syllables in contrast to less frequent syllables, reducing them in their duration. The need to reduce effort arises independently of what language is produced. Accordingly, practitioners shorten glossolalia syllables when they are frequent in German as well as when they are frequent in glossolalia.

Above, we have argued that probability effects in glossolalia are best explained by some type of frequency-sensitive representation of syllabic motor programs. While we have focused here on instances of speech production devoid of any meaning, our main goal is nevertheless to understand the more general relationship between probability and phonetic variation in speech production. Accordingly, we now turn to the implications of the present findings for speech production in natural languages—that is, when speakers do need semantics to initiate the production process.

As we have argued above, various proposals have suggested different stages at which probability affects speech preparation, among them the stage of lexical access (Bell et al., 2009; Levelt et al., 1999), the stage of syllable access (Cholin et al., 2006), the stage of gestural representation (Tomaschek et al., 2018), and during the communication process itself in relation to information (Aylett & Turk, 2004). The present findings for glossolalia support the idea that probability effects can be located at the level of syllable access and representation. This implies that probability effects do not function at just one stage of speech production; instead, they are distributed across all stages, from lexical access until the kinematic process of articulation itself.

## Appendix

In addition to exhibiting differences in statistical properties, glossolalia and German syllable sets also exhibit different phonotactic properties. Syllables in all *n*-gram sequences in Table 2 largely exhibit a CV structure. By contrast, German allows closed syllables with very complex onsets and codas (Wiese, 1988). In the entire glossolalia corpus, we find CV syllables 7,162 times, CVC 373 times, V 21 times, C 10 times, VCV 9 times, and VC 3 times.^3^ Inspection of the *n*-gram sequences also supports the hypothesis that glossolalia does not consist of words borrowed from the speakers’ native language. Instead, it seems as though the community has developed a set of independent but similar glossolalia patterns. For example, Speaker 1 produced [sɔkɔtara], Speaker 8 [ʃIkətʊrʊ], and Speaker 10 [sɔkʊtʊrʊ]. Speaker 2 produced [ʃIkerIa], Speaker 3 [səkɔra], Speaker 4 [ʃɔkʊrIa], and Speaker 7 [ʃekara]. While these utterances are not exactly the same across all speakers, the first three speakers exhibit the onset pattern [s/ ʃ] -[k]-[t]-[r] and the others exhibit the onset pattern [s/ʃ] -[k]-[r].

Table A1 illustrates that these patterns are present in the entire data set. The simple onset patterns [n]-[n], [n]-[n]-[n], and [n]-[n]-[n]-[n] are the most frequent. Note that although there are also frequent patterns which do not use a single consonant but a combination of several consonants, such as [n]-[j]-[s], [k]-[t]-[r], [s]-[k]-[t]-[r], and so on, the majority of syllable patterns are based on one consonant, such as [n], [r], or [l] .

**Table A1.** Ten Most Frequent Onset Patterns and Their Number of Observations.

1 gram	Fr1	2 gram	Fr2	3 gram	Fr3	4 gram	Fr4	5 gram	Fr5
N	1,274	n n	455	n n n	178	n n n n	58	s k t r v	31
R	1,022	k r	200	r r r	105	s k t r	58	r r r r r	31
d	672	r n	194	k t r	80	r r r r	58	d s k t r	29
k	658	r r	192	v xn d	76	r v xn d	45	t r v xn d	28
l	598	l l	189	l l l	73	r n j s	34	k t r v xn	27
s	565	d d	172	n j s	70	v xn d s	31	n j s t r	25
x	357	t r	150	s k t	68	k t r v	31	xn d s k t	21
S	342	s k	141	r n n	59	j s t r	30	v xn d s k	21
j	306	n j	126	r b S	53	d s k t	30	n n n n n	18
b	289	k t	116	d d d	52	t r v xn	29	r n n n n	17

## Notes

1. All data and analyses will be made available upon publication.

2. An anonymous reviewer suggested testing the degree to which the present findings may also be due to reduplication. We found that across speakers, an average of 11.6% (*SD* = 7.9% *P* with min = 2% and max = 26%) of our data consists of reduplication. The distribution of percentage of reduplication was relatively normal. We tested the effect of reduplication by fitting syllable duration with P(S0) based on the entire glossolalia corpus plus a binary predictor stating whether or not a syllable is repeated in a sequence. We found that, as the reviewer suggested, reduplicated syllables were significantly shorter than those that were not (estimate = –0.343, standard error = 0.0184, *t* = –18.63). However, the effect of syllable probability was still significant (estimate = –0.0698, standard error = 0.0058, *t* = –11.95), which indicates that the frequency effect exists on top of the reduplication effect.

3. For the sake of simplicity, we collapsed CC and CCC into C, as CC and CCC occurred only 148 times as an onset and two times as a coda.
